# Anthropometric Profile and Position-Specific Changes in Segmental Body Composition of Professional Football Players Throughout a Training Period

**DOI:** 10.3390/sports12100285

**Published:** 2024-10-21

**Authors:** Wiktoria Staśkiewicz-Bartecka, Karolina Krupa-Kotara, Mateusz Rozmiarek, Ewa Malchrowicz-Mośko, Mateusz Grajek, Saioa Agirre Elordui, Jokin Urriolabeitia Razkin, Arkaitz Castañeda Babarro

**Affiliations:** 1 Department of Food Technology and Quality Evaluation, Department of Dietetics, Faculty of Public Health in Bytom, Medical University of Silesia in Katowice, 41-808 Zabrze, Poland; wstaskiewicz@sum.edu.pl; 2 Department of Epidemiology, Faculty of Public Health in Bytom, Medical University of Silesia in Katowice, 41-902 Bytom, Poland; kkrupa@sum.edu.pl; 3 Department of Sports Tourism, Faculty of Physical Culture Sciences, Poznan University of Physical Education, 61-871 Poznan, Poland; rozmiarek@awf.poznan.pl (M.R.); malchrowicz@awf.poznan.pl (E.M.-M.); 4 Department of Public Health, Faculty of Public Health in Bytom, Medical University of Silesia in Katowice, 41-902 Bytom, Poland; mgrajek@sum.edu.pl; 5 Department of Physical Activity and Sports Sciences, Faculty of Education and Sports, University of Deusto, 48007 Bilbao, Spain; s.agirre@deusto.es (S.A.E.); jokin.u@deusto.es (J.U.R.)

**Keywords:** anthropometric profiling, body composition, football players, field position, macrocycle

## Abstract

Body and anthropometric profiles of football players vary depending on the physiological and technical demands imposed by different positions. The aim of this study was to evaluate the body composition of professional soccer players in relation to their position on the field during a training macrocycle. The Direct Segmental Multi-Frequency Bioelectrical Impedance Analysis method was used to analyze 58 players at six key moments of the macrocycle. The results show that body profiles are adjusted to the specific demands of each position. Midfielders showed the lowest muscle mass, while defenders showed many notable changes. In general, as the season progressed, all field players experienced an increase in trunk body fat. Fat and lean mass values of goalkeepers differed greatly from the rest. The greatest variations in body composition were observed during pre-season and transition in relation to variations in training load and competitive intensity. The results suggest that the phase of the macrocycle has a greater influence on these variations, although the physical characteristics of each position are relevant. Understanding these dynamics allows for the design of more personalized and efficient training programs to optimize the performance of footballers according to their roles and each stage of the season.

## 1. Introduction

Soccer can be characterized as a primarily low to moderate-intensity activity interspersed with periods of high intensity [1]. In recent years, the physical demands in elite soccer matches have increased significantly, and soccer players have to adapt to these [2]. Body composition, physiological and physical abilities, and other determinants of optimal performance can be easily measured, allowing the various physical skills necessary for success in the sport to be assessed and developed [3,4].

In soccer, it is common practice to evaluate the anthropometric profile of players in order to optimize physical performance and reduce injuries [5]. It should be noted that adequate levels of fat mass allow players to move better and perform better during training and matches [6]. In addition, lean mass, especially muscle mass, should be controlled because it can affect some performance factors such as speed, strength, power, and injury risk [7]. In this regard, the playing position is crucial since differences in the physiological and metabolic needs of each position may indicate the presence of different anthropometric and body composition characteristics [8].

Soccer players are subjected to technical and tactical actions depending on their position on the field [9]. In this sport, soccer players are generally classified into four positions: goalkeepers, defenders, midfielders, and forwards (strikers). In addition, the role played on the field is essential since the physiological and metabolic demands of each position can influence the anthropometric (body mass, height, BMI) and body composition characteristics of each player [10,11]. Previous studies have shown that certain physical and anthropometric attributes predispose certain players to success in soccer [8]. This indicates that these variables point to morphological optimization within soccer and that anthropometric measurements of players should be an integral part of a performance profiling program [8]. For example, to improve the performance of each player, optimizing the development of the specific qualities required in each case, professionals could design individualized training programs based on detailed knowledge of the anthropometric, physiological, and physical characteristics of the players, depending on their competitive level, playing position and age [3]. Body composition assessments in soccer players are essential to improve performance and evaluate the effectiveness of the implemented training regimen, which is a crucial component of personalized and periodized training programs for athletes [4]. Tracking body fat is particularly important, as maintaining adequate levels allows players to move more efficiently during training and matches [6]. Similarly, tracking lean mass (especially muscle mass) is vital because inadequate training loads, whether too high or too low, can lead to unfavorable physiological changes. These changes can negatively impact key performance parameters such as power, strength, speed, and injury risk.

On the other hand, soccer season planning is generally divided into three phases: the pre-competitive, competitive, and transitional periods [12]. The pre-competitive period focuses on developing the necessary skills for the competitive phase [13]. It can also be known as the preparatory period because it focuses on rebuilding the players’ fitness after the transition phase [14]. In the competitive period, loads should be adjusted to optimize physiological adaptations while avoiding overtraining and injuries [15]. It is characterized by seeking to improve sports performance based on the skills and performance acquired in the previous phase. In the last transition phase, training is significantly reduced. Therefore, it is the right time for athletes to recover for the next season [12]. It was found that the body composition of elite soccer players varies greatly depending on their playing position and the time of the season [16]. Furthermore, throughout the season, it is essential to consider the aforementioned variables, as they can have an impact on training and performance during competition [1]. Therefore, regular measurements of body composition can help players to stay at their best.

The aim of this study was to evaluate the body composition of professional soccer players in relation to their position on the field during a training macrocycle.

## 2. Materials and Methods

### 2.1. Study Design

The study was conducted between 7 January and 23 July 2021, covering the spring round of the 2020–2021 PKO BP Ekstraklasa season, Poland’s premier football league. The participants were male footballers from two Silesian clubs competing in the PKO BP Ekstraklasa during the 2020/2021 season. This paper builds upon prior research with the methodology outlined in previous works [17,18].

The inclusion criteria required participants to be professional footballers (with a signed professional contract) from one of the involved clubs, willing to participate, and free of long-term injuries that would prevent them from training or playing for the entire seven-month study. Exclusion criteria included transfers between teams during the study period, absence from training or matches for 14 days or more due to illness, injury, isolation, or quarantine related to the COVID-19 pandemic, or failure to participate in at least one of the six scheduled measurements for reasons other than those listed [17,18].

The study adhered to the principles of the World Medical Association’s Declaration of Helsinki. The bioethics committee of the Silesian Medical University in Katowice reviewed and approved the study protocol (PCN/0022/KB/68/I/20). All participants provided written informed consent.

Six body composition assessments were conducted during the pre-competition, competition, and transition phases of the 2020/2021 PKO BP Ekstraklasa spring season. Measurement scheduling was coordinated with league and cup competitions, ensuring sufficient post-exercise recovery time (per the guidelines provided by InBody, a minimum of 24 h was maintained between the last physical activity and body composition analysis). Measurements were taken at key points in the macrocycle as previously defined: (1) the first measurement occurred before the preparatory phase, (2) the second after the preparatory phase, (3) the third, and (4) the fourth during the competition phase, (5) the fifth after the competition phase, and (6) the sixth following the transition phase [17,18].

### 2.2. Subjects

In this investigation, a cohort of 58 professional football players, aged between 18 and 31 years, was recruited. The study sample included athletes of various nationalities, namely Polish, Slovakian, Spanish, Portuguese, Greek, Slovenian, Czech, Austrian, Danish, Hungarian, Ghanaian, and Gambian origin. The participants were categorized according to their specific positional roles on the field, which were classified as strikers, midfielders, defenders, and goalkeepers. Finally, due to the fact that some participants were not able to perform all the measurements, a total of 38 athletes contributed 228 body composition measurements, with strict adherence to the predetermined inclusion and exclusion criteria to ensure the validity and reliability of the data collected.

### 2.3. Methodology

Before the initial body composition assessment, participants’ height was measured. Body mass was recorded at each subsequent body composition measurement. Height (in centimeters) and body mass (in kilograms) were measured with a precision of 0.1 cm and 0.1 kg, respectively, using SECA 756 equipment (Seca GmbH & Co. KG, Hamburg, Germany). The InBody 770 device (InBody USA, Cerritos, CA, USA) was used to determine body mass, and the Body Mass Index (BMI) was subsequently calculated by dividing body mass (kg) by the square of the height (m). The resulting BMI values were utilized to compare height-to-weight ratios with WHO recommendations and European population standards [19].

Body composition was evaluated using the Direct Segmental Multi-Frequency Bioelectrical Impedance Analysis (DSM-BIA) method (InBody 770, InBody USA, Cerritos, CA, USA). The DSM-BIA method, based on the concept that the human body consists of five interconnected cylindrical segments, directly measures impedance from specific internal compartments. A tetrapolar eight-point touch electrode system was employed to measure impedance across the torso, arms, and legs at six different frequencies (1 kHz, 5 kHz, 50 kHz, 250 kHz, 500 kHz, and 1000 kHz), with the analyzer operating at a current of 80 µA [20,21].

Anthropometric measurements were further analyzed according to lateralization based on participants’ self-reported dominant or assisting limb. The dominant limb was identified as the one with superior strength and motor abilities. Through DSM-BIA, regional fat mass (arms, legs, and trunk), lean body mass (measured segmentally for arms, legs, and trunk), and visceral fat level were assessed.

Body composition parameters were determined using Lookin’Body Software version 120.3.0.6, following the manufacturer’s established guidelines and methodology. Before each measurement session, the analyzer was calibrated using a circuit with known impedance values (resistance = 500.0; reactance = 0.1; error = 0.9%). The entire procedure adhered to established protocols and relevant literature [20,21,22].

### 2.4. Statistical Analysis

The data were processed using Statistica 13.3 software (StatSoft Polska Sp. z o.o., Cracow, Poland) and the R 4.0.0 statistical package (2020) (The R Foundation for Statistical Computing, Vienna, Austria), in compliance with the GNU GPL license. Results were expressed as mean values accompanied by their standard deviations.

The normality of the data distribution was assessed via the Shapiro-Wilk test. Differences between group means, categorized by player position (goalkeeper, defender, midfielder, striker), were evaluated through Analysis of Variance (ANOVA). For distributions that deviated from normality, the Kruskal-Wallis test was employed to assess concordance. Post-hoc comparisons of group means were conducted using Tukey’s HSD test for parametric data and Dunn’s test for non-parametric data. The average of the six body composition measurements across the entire research period, as well as individual measurements, were calculated.

To compare anthropometric data collected at different time points, ANOVA for repeated measures or the non-parametric Friedman test was utilized based on the distribution’s adherence to normality. Post-hoc intergroup analyses employed either Tukey’s HSD test or the post-hoc test following Friedman’s test.

A three-factor ANOVA was implemented to analyze the composite data related to anthropometric profiling and segmental body composition changes in professional football players, categorized by position on the field, over the course of the training macrocycle. This approach enabled an evaluation of statistically significant differences between groups, considering various influencing factors and their interactions. Specifically, the three-factor ANOVA was chosen to account for the following variables:

Position on the Field: Anthropometric characteristics and body composition can differ based on a player’s role on the field. Analyzing these differences helps identify trends or variations in these parameters across groups.

Timing of the Macrocycle Phases: The training macrocycle for professional football players includes several phases, such as preparatory, competitive, and transition periods. Body composition and anthropometric parameters may fluctuate depending on the intensity and type of training in each phase. This factor determined whether significant changes occurred across the macrocycle phases.

Limb Comparison: Segmental body composition analyzes the differences between body parts, such as upper versus lower limbs. These variations are critical for assessing strength, function, and overall performance. The limb comparison factor assessed potential differences in body composition between different regions of the body.

The three-factor ANOVA thus facilitated the investigation of the effects of these three variables—position, timing, and limb comparison—on the anthropometric and body composition metrics of professional footballers. Furthermore, corrections were applied for multiple comparisons and small subgroup statistical analyses.

The criterion for statistical significance was *p* < 0.05.

## 3. Results

Table 1 shows the characteristics of the study group.

Statistically significant differences were found in the content of lean body mass in body parts of players performing various functions on the pitch. Significantly lower values of lean body mass concerned midfielders (*p* < 0.05) (Table 2).

There was a statistically significant difference in the content of lean body mass between measurements 1–6 in the study group (Table 3). In the group of strikers, a decrease in the content of lean body mass in the assisting leg was found between measurements 2 and 5 (*p* < 0.05). In the group of midfielders, there was a decrease in lean body mass in the dominant arm between the 2nd and 3rd measurements (*p* < 0.05). In the group of defenders, there was an increase in the content of lean body mass in the dominant leg between the 1st and 2nd measurement, a decrease in the content between the 2nd and 5th measurement (*p* < 0.01), and an increase in the content of lean body mass in the assisting leg between the 1st and 6th, and 2nd and 6th measurement. (*p* < 0.01).

There was no difference in the content of fat tissue in the limbs depending on the position on the pitch (Table 4).

Statistically significant differences in fat tissue content in the players’ body parts in measurements 1–6 were demonstrated (Table 5). The mass of adipose tissue in the dominant arm increased in the goalkeeper group between the 4th and 6th measurements (*p* < 0.05). An increase in the mass of fat tissue in the trunk was observed in the group of midfielders and defenders between the 2nd and 6th measurements (*p* < 0.001; *p* < 0.01). An increase in fat tissue mass in the dominant and assisting lower limb was demonstrated in defenders between the 2nd, 3rd, and 6th measurements (*p* < 0.01; *p* < 0.01).

## 4. Discussion

Soccer players require certain specific physiological and technical demands depending on their position on the field [23]. The body composition and anthropometric profiles of players may vary according to the role they play [24]. Factors such as the period of the season can influence these variations.

The purpose of this research was to assess the body composition of professional soccer players based on their positioning on the field throughout a training macrocycle.

The results of the present study show significant variations in the body composition of professional soccer players throughout the season.

Midfielders showed significantly lower values for lean body mass. This may be closely linked to the fact that midfielders travel the longest distances, around 11 km per match, and have lower weight, height, and total and segmental muscle mass compared to the other roles [7,23,24,25].

Differences in lean mass and adipose tissue content were found between the first and last measurements. Previous studies support these significant differences with respect to the first and last measurements in relation to body fat and fat-free mass [8,23]. This may be mainly due to the specific characteristics of each phase.

The defenders showed a greater number of significant changes during the measurements. An increase in the lean mass of the dominant leg was observed between the preseason and the competitive phase, followed by a reduction at the beginning of the transition phase. These results may be affected by the intensity and volume of physical work during the preparatory and competitive phase, which favors hypertrophy, while the transition phase reduces the load, leading to loss of muscle mass [12,26].

However, adipose tissue in both legs increased in the transition period compared to the second and third measurements. This suggests that decreased physical activity and possible variations in diet during transition contribute to the increase in adipose tissue [27].

Of note is the increase of fat tissue mass in the trunk experienced by defenders and midfielders from the second to the sixth measurement. Variations in the intensity of these specific positions, the type of training, and the breaks throughout these phases of the season could cause this change [25,27].

On the other hand, at the end of the season, the goalkeepers experienced an increase in adipose tissue in their dominant arm. This could be related to a different activity profile since goalkeepers tend to make fewer movements during matches, which can be translated as a lower physical demand compared to the other positions; therefore, it is possible that their training does not compensate for their caloric balance, especially in the transition phase [6].

As for the forwards, a significant decrease in the lean mass of the auxiliary leg was shown between the second and fifth measurements. This result may be linked to the reduction of the training load and the intensity of the matches during transition, similar to what happens with the defenders [9].

The physical demands and training loads vary according to the different stages of the season and the different positions on the field. In the present study, most of the changes in the body composition of the players occurred during the preseason and the transition phase. On the other hand, scientific evidence supports that the fat and lean mass percentage values of goalkeepers differ greatly from the rest of the positions on the field, although, in general, there are very few differences among field players [7,8,9,23,28,29]. For this reason, it is suggested that variations in body composition may be more influenced by the period of the season than by the specific role of each player.

The results of this study show both similarities and notable variances with those of other studies on the body composition of professional soccer players. For example, Carling and Orhant reported considerable variations in muscle mass and body fat of elite players throughout the season, particularly between the preseason and transition phases, which coincides with the fluctuations observed in the group of defenders and midfielders in this study [8]. The results of this study, however, indicate a more marked increase in trunk fat, particularly during the transition phase, whereas the studies by Barnes et al. and Milanese et al. found less fat accumulation in elite players [2,28]. This suggests that variations in training planning and access to nutritional resources between competitive levels may have a significant impact on these changes. Furthermore, studies like Cárdenas-Fernández et al. and Slimani et al. showed that players at higher levels typically maintain more stability in their body composition throughout the season, which was not as noticeable in the sample under study [9,13]. These discrepancies may be explained by the higher tactical and physical demands placed on professional athletes as well as the advanced training programs they are subjected to. However, the results of the study also suggest that some of these variations may be consistent regardless of the level of competition, underscoring the influence of the training cycle on body composition.

Despite the importance of body composition assessments in football players, there are important restrictions to take into account. Firstly, the lack of physiological parameters, such as heart rate and blood markers, restricts a complete understanding of players’ fitness and performance. For instance, studies have shown that an athlete’s heart rate response during vigorous exercise can offer valuable information about the athlete’s cardiovascular condition [30]. Similarly, recent research has shown that submaximal heart rate is a useful tool for tracking changes in cardiorespiratory fitness [31]. Likewise, the measurement of blood markers, such as lactate, is essential to evaluate performance and fatigue in a football setting [32,33]. Additionally, the absence of football-specific performance tests, such as change-of-direction ability and cardiorespiratory fitness, limits the ability to assess the actual physical demands of the game. The concordance between dribbling and change-of-direction deficits has been recognized as a crucial sign of directional asymmetry in young footballers [34]. This underlines the importance of incorporating specific tests that capture the demands of the sport in order to achieve a more complete evaluation of player performance. Consequently, including these elements in assessments may provide a more accurate view of footballers’ physical fitness.

## 5. Conclusions

The results of this study have practical implications for professionals in this field. Understanding changes in body composition according to position and their evolution throughout the training macrocycle allows these professionals to design more specific and effective training and nutrition programs. Regular monitoring of body composition makes it easier to adjust training loads and nutritional intake on an individualized basis, thus optimizing performance and reducing the risk of injury caused by muscle imbalances or an undue proportion of fat mass.

The study reveals that variations in the body composition of soccer players are influenced by the position they occupy on the field and the stage of the season. The body profiles of midfielders, defenders, strikers, and goalkeepers are adapted to the specific demands of each role. However, it is during the preseason and transition phase when the body composition of players changes the most due to changes in training load and competitive intensity. Although the physical characteristics of each position are important, it seems that the season has a greater impact on these variations.

## Figures and Tables

**Table 1:** Characteristics of the study group (n = 38).

Variable		Age [Years] X ± S Med (Min–Max)	Height [cm] X ± S Med (Min–Max)	Body Mass [kg] X ± S Med (Min–Max)	BMI [kg/m^2^] X ± S Med (Min–Max)
Field position	Striker	25.5 ± 5.89 24.5 (21–28)	182.5 ± 5.32 184.5 (176–185)	79.7 ± 7.55 80.55 (74.17–85.23)	23.96 ± 1.25 24.37 (22.72–24.91)
	Midfielder	24.13 ± 4.69 24 (20–26)	179.63 ± 4.6 177 (176–183)	75.1 ± 6.48 72.88 (71.08–76.07)	23.24 ± 1.24 23.26 (22.23–23.8)
	Defender	27.5 ± 4.5 28 (26.5–30)	183.88 ± 5.11 183.5 (180.5–187.5)	83.34 ± 5.71 82.62 (78.59–86.91)	24.63 ± 0.85 24.63 (23.96–25.12)
	Goalkeeper	27.8 ± 7.19 27 (26–28)	188.5 ± 3.5 187 (186–192)	83.35 ± 5.6 81.83 (80.15–86.07)	23.45 ± 1.3 23.65 (22.54–24.61)
	*p*-value	0.32; NS	0.01; M vs. G **	0.01; P vs. D **	0.03; M vs. D *

X = average; SD = standard deviation; Med = median; Min = minimum; Max = maximum; * = *p* < 0.05; ** = *p* < 0.01.

**Table 2:** Fat-free mass content of the players’ body components.

Variable		Fat-Free Mass [kg]				
		Dominant Arm X ± SD Med (Min–Max)	Assisting Arm X ± SD Med (Min–Max)	Trunk X ± SD Med (Min–Max)	Dominant Leg X ± SD Med (Min–Max)	Assisting Leg X ± SD Med (Min–Max)
Total		4.07 ± 0.42_4.07 (3.8–4.31)	4.05 ± 0.42_4.02 (3.69–4.32)	30.69 ± 2.41_30.53 (28.95–32.37)	11.16 ± 1.06_10.99 (10.31–11.99)	11.08 ± 1.01_10.99 (10.25–11.81)
Field position	Striker	4.02 ± 0.34_4.09 (3.66–4.33)	4.02 ± 0.34_4.09 (3.66–4.33)	30.46 ± 2.13_30.97 (28.45–32.37)	11.34 ± 1.21_11.43 (10.49–12.26)	11.34 ± 1.22_11.35 (10.58–12.05)
	Midfielder	3.83 ± 0.44_3.69 (3.51–4.03) *	3.83 ± 0.44_3.69 (3.51–4.03) *	29.34 ± 2.48_29.08 (27.58–30.18) *	10.57 ± 0.91_10.3 (9.79–11.24)	10.49 ± 0.88_10.22 (9.79–11.12) *
	Defender	4.22 ± 0.35_4.11 (3.98–4.48) *	4.22 ± 0.35_4.11 (3.98–4.48) *	31.73 ± 1.91_31.22 (30.3–33.27) *	11.61 ± 0.97_11.29 (10.78–12.55)	11.44 ± 0.85_11.28 (10.7–12.3) *
	Goalkeeper	4.35 ± 0.26_4.32 (4.11–4.51) *	4.35 ± 0.26_4.32 (4.11–4.51) *	32.48 ± 1.55_32.93 (31.45–33.15)	11.61 ± 0.96_11.99 (10.94–11.99)	11.68 ± 0.82_11.81 (11.43–12.03)
	*p*-value	0.007; M vs. D * M vs. G *	0.008; M vs. D * M vs. G *	0.007; M vs. D * M vs. G *	0.04; M vs. D *	0.02; M vs. D *

X = average; SD = standard deviation; Med = median; Min = minimum; Max = maximum; * = *p* < 0.05.

**Table 3:** Changes in fat-free mass in body parts of football players regarding field position.

Variable	Measurement	Striker (S) X ± SD Med (Min–Max)	*p*-Value	Midfielder (M) X ± SD Med (Min–Max)	*p*-Value	Defender (D) X ± SD Med (Min–Max)	*p*-Value	Goalkeeper (G) X ± SD Med (Min–Max)	*p*-Value	Position *p*-Value
Dominant arm	1	3.99 ± 0.36_4.04 (3.6–4.35)	0.37; NS	3.86 ± 0.44_3.84 (3.54–4.08)	0.03; 2 vs. 3 *	4.23 ± 0.3_4.21 (4–4.46)	0.15; NS	4.34 ± 0.25_4.24 (4.17–4.53)	0.42; NS	0.03; *p* < 0.05 M vs. D *
	2	4.02 ± 0.39_4.1 (3.64–4.33)		3.88 ± 0.44_3.89 (3.59–4.02)		4.29 ± 0.27_4.22 (4.13–4.42)		4.41 ± 0.3_4.3 (4.22–4.61)		0.01; *p* < 0.05 M vs. D * M vs. G *
	3	4.01 ± 0.37_4.11 (3.74–4.19)		3.85 ± 0.43_3.81 (3.54–4.02)		4.23 ± 0.31_4.16 (4.03–4.43)		4.39 ± 0.27_4.32 (4.27–4.6)		0.01; *p* < 0.05 M vs. D * M vs. G *
	4	3.97 ± 0.35_4.04 (3.72–4.15)		3.85 ± 0.44_3.69 (3.55–4.11)		4.24 ± 0.33_4.11 (4.03–4.58)		4.44 ± 0.28_4.51 (4.32–4.6)		0.01; *p* < 0.05
	5	3.98 ± 0.35_4.08 (3.62–4.16)		3.83 ± 0.48_3.71 (3.53–3.98)		4.24 ± 0.32_4.15 (4.04–4.62)		4.4 ± 0.3_4.46 (4.16–4.58)		M vs. G *
	6	4.03 ± 0.39_4.13 (3.64–4.27)		3.86 ± 0.48_3.68 (3.56–4.06)		4.28 ± 0.34_4.15 (4.05–4.66)		4.43 ± 0.29_4.51 (4.14–4.66)		0.008; *p* < 0.01 M vs. D * M vs. G *
Assisting arm	1	4.02 ± 0.36_4.07 (3.64–4.29)	0.29; NS	3.81 ± 0.43_3.74 (3.49–4)	0.08; NS	4.2 ± 0.34_4.16 (3.94–4.41)	0.16; NS	4.27 ± 0.24_4.14 (4.09–4.49)	0.2; NS	0.02; *p* < 0.05 M vs. D * M vs. G *
	2	4.01 ± 0.37_4.11 (3.6–4.25)		3.85 ± 0.46_3.74 (3.51–4.03)		4.25 ± 0.32_4.17 (4.06–4.4)		4.32 ± 0.28_4.2 (4.11–4.56)		0.007; *p* < 0.01 M vs. D *
	3	4 ± 0.32_4.11 (3.69–4.28)		3.85 ± 0.43_3.73 (3.54–4.02)		4.21 ± 0.34_4.17 (3.96–4.39)		4.32 ± 0.26_4.18 (4.17–4.52)		0.02; *p* < 0.05 M vs. D *
	4	3.97 ± 0.32_4.05 (3.67–4.21)		3.82 ± 0.45_3.76 (3.52–4.01)		4.22 ± 0.37_4.1 (3.96–4.54)		4.38 ± 0.26_4.45 (4.19–4.5)		0.008; *p* < 0.01 M vs. D * M vs. G *
	5	4.02 ± 0.32_4.07 (3.7–4.32)		3.79 ± 0.44_3.61 (3.5–4)		4.21 ± 0.37_4.1 (3.94–4.57)		4.35 ± 0.3_4.38 (4.07–4.46)		0.006; *p* < 0.01 M vs. D * M vs. G *
	6	4.07 ± 0.36_4.14 (3.69–4.35)		3.83 ± 0.45_3.68 (3.49–4.01)		4.24 ± 0.39_4.14 (3.92–4.62)		4.44 ± 0.3_4.53 (4.21–4.63)		0.007; *p* < 0.01 M vs. D * M vs. G *
Trunk	1	30.45 ± 2.12_30.8 (28.2–32.4)	0.29; NS	29.32 ± 2.47_29.3 (27.6–30.2)	0.23; NS	31.53 ± 1.95_31 (30.25–32.9)	0.06; NS	32.16 ± 1.43_31.9 (31.2–33.2)	0.34; NS	0.01; *p* < 0.05 M vs. G *
	2	30.42 ± 2.26_30.85 (28.2–32.5)		29.45 ± 2.52_29.4 (27.7–30.2)		31.9 ± 1.74_31.5 (30.9–32.8)		32.38 ± 1.65_32.4 (31.4–33.2)		0.007; *p* < 0.01 M vs. D * M vs. G *
	3	30.48 ± 2.12_31.1 (28.7–32.1)		29.4 ± 2.42_29.2 (27.5–30.2)		31.69 ± 1.92_31.45 (30.2–33)		32.38 ± 1.48_32.4 (32–33.1)		0.01; *p* < 0.05 M vs. D * M vs. G *
	4	30.28 ± 2.1_30.75 (28.6–31.8)		29.33 ± 2.5_28.6 (27.6–30.4)		31.69 ± 1.97_30.95 (30.35–33.45)		32.68 ± 1.71_33.1 (32–33.7)		0.008; *p* < 0.01 M vs. D * M vs. G *
	5	30.48 ± 2.03_31.1 (28.5–32.2)		29.22 ± 2.53_28.6 (27.5–30.3)		31.72 ± 2_31.25 (30.25–33.6)		32.5 ± 1.82_33.1 (31.1–33.4)		0.007; *p* < 0.01 M vs. D * M vs. G *
	6	30.67 ± 2.22_31.2 (28.5–32.5)		29.33 ± 2.53_28.6 (27.5–30.4)		31.87 ± 2.08_31.7 (30.15–33.85)		32.8 ± 1.55_33.2 (31.4–33.8)		0.008; *p* < 0.01 M vs. D * M vs. G *
Dominant leg	1	11.29 ± 1.19_11.34 (10.45–12.02)	0.07; NS	10.49 ± 0.97_10.29 (9.52–11.18)	0.07; NS	11.5 ± 0.94_11.16 (10.61–12.55)	0.001; 1 vs. 2 ** 2 vs. 5 **	11.52 ± 0.93_11.75 (10.8–11.92)	0.63; NS	0.05; *p* < 0.05 M vs. D *
	2	11.4 ± 1.24_11.5 (10.57–12.32)		10.58 ± 0.93_10.37 (9.75–11.29)		11.73 ± 1.01_11.46 (10.91–12.66)		11.6 ± 0.9_11.84 (11.01–12.11)		0.03; *p* < 0.05 M vs. D *
	3	11.39 ± 1.18_11.49 (10.61–12.34)		10.6 ± 0.9_10.34 (9.91–11.26)		11.63 ± 0.98_11.41 (10.77–12.63)		11.62 ± 0.95_11.89 (10.94–12.29)		0.04; *p* < 0.05 M vs. D *
	4	11.39 ± 1.16_11.49 (10.58–12.29)		10.63 ± 0.93_10.44 (9.92–11.22)		11.63 ± 0.94_11.33 (10.93–12.53)		11.67 ± 1.05_12.08 (10.91–12.1)		0.05; NS
	5	11.25 ± 1.25_11.32 (10.28–12.27)		10.51 ± 0.9_10.19 (9.82–11.19)		11.53 ± 0.96_11.17 (10.84–12.42)		11.59 ± 0.99_11.78 (10.88–12.17)		0.04; *p* < 0.05 M vs. D *
	6	11.35 ± 1.28_11.44 (10.46–12.34)		10.6 ± 0.9_10.13 (9.92–11.32)		11.64 ± 1.05_11.26 (10.76–12.54)		11.68 ± 0.97_11.72 (11.11–12.19)		0.05; NS
Assisting leg	1	11.29 ± 1.27_11.26 (10.52–11.83)	0.01; 2 vs. 5 *	10.42 ± 0.93_10.32 (9.47–11.03)	0.05; NS	11.34 ± 0.86_11.12 (10.53–12.21)	0.007; 1 vs. 2 * 1 vs. 6 *	11.64 ± 0.82_11.58 (11.45–12.02)	0.94; NS	0.03; *p* < 0.05 M vs. D * M vs. G *
	2	11.41 ± 1.25_11.44 (10.68–12.07)		10.5 ± 0.9_10.36 (9.91–11.14)		11.53 ± 0.86_11.51 (10.83–12.38)		11.68 ± 0.78_11.78 (11.54–12.1)		0.02; *p* < 0.05 M vs. D *
	3	11.38 ± 1.2_11.43 (10.65–12.09)		10.53 ± 0.84_10.3 (9.9–11.11)		11.45 ± 0.84_11.4 (10.66–12.33)		11.68 ± 0.8_11.7 (11.43–12.3)		0.03; *p* < 0.05 M vs. D *
	4	11.35 ± 1.16_11.41 (10.62–12.07)		10.52 ± 0.88_10.33 (9.84–11.08)		11.44 ± 0.82_11.27 (10.8–12.26)		11.71 ± 0.88_11.91 (11.37–12.13)		0.02; *p* < 0.05 M vs. D *
	5	11.24 ± 1.22_11.23 (10.44–12.03)		10.43 ± 0.88_10.2 (9.81–11.05)		11.38 ± 0.82_11.19 (10.76–12.17)		11.65 ± 0.83_11.85 (11.33–11.99)		0.02; *p* < 0.05 M vs. D *
	6	11.38 ± 1.26_11.37 (10.59–12.21)		10.51 ± 0.9_10.14 (9.8–11.27)		11.53 ± 0.93_11.23 (10.76–12.43)		11.71 ± 0.83_11.79 (11.45–11.89)		0.03; *p* < 0.05 M vs. D *

X = average; SD = standard deviation; Med = median; Min = minimum; Max = maximum; * = *p* < 0.05; ** = *p* < 0.01.

**Table 4:** Fat mass content of the players’ body components.

Variable		Body Fat Mass [kg]					
		Dominant Arm X ± SD Med (Min–Max)	Assisting Arm X ± SD Med (Min–Max)	Trunk X ± SD Med (Min–Max)	Dominant Leg X ± SD Med (Min–Max)	Assisting Leg X ± SD Med (Min–Max)	Visceral Fat Level X ± SD Med (Min–Max)
Total		0.24 ± 0.13_0.19 (0.13–0.38)	0.25 ± 0.13_0.2 (0.15–0.37)	4.07 ± 1.52_3.66 (2.9–5.27)	1.31 ± 0.31_1.23 (1.12–1.53)	1.3 ± 0.31_1.23 (1.12–1.52)	2.29 ± 1.21_2 (1.33–3.33)
Field Position	Striker	0.25 ± 0.1_0.23 (0.18–0.33)	0.24 ± 0.08_0.2 (0.18–0.32)	3.99 ± 1.04_3.9 (2.93–5)	1.33 ± 0.23_1.32 (1.13–1.53)	1.32 ± 0.23_1.31 (1.13–1.52)	2 ± 0.74_2 (1.5–2.33)
	Midfielder	0.2 ± 0.11_0.18 (0.1–0.22)	0.21 ± 0.1_0.2 (0.12–0.23)	3.36 ± 1.22_3.1 (2.47–3.68)	1.19 ± 0.25_1.15 (1.03–1.3)	1.19 ± 0.25_1.15 (1.02–1.3)	1.8 ± 1.05_1.33 (1–2)
	Defender	0.29 ± 0.15_0.37 (0.13–0.41)	0.29 ± 0.15_0.36 (0.14–0.41)	4.69 ± 1.77_5.2 (3.49–6.18)	1.38 ± 0.38_1.49 (1.13–1.73)	1.36 ± 0.37_1.48 (1.13–1.67)	2.89 ± 1.37_3.25 (1.67–4.08)
	Goalkeeper	0.22 ± 0.18_0.15 (0.13–0.17)	0.26 ± 0.2_0.18 (0.15–0.23)	4.84 ± 1.53_4.32 (3.92–4.9)	1.45 ± 0.37_1.37 (1.23–1.43)	1.44 ± 0.37_1.37 (1.23–1.4)	2.7 ± 1.25_2.33 (2.17–3)
	*p*-value	0.49	0.59	0.08	0.31	0.32	0.1

X = average; SD = standard deviation; Med = median; Min = minimum; Max = maximum.

**Table 5:** Changes in fat mass in body parts and visceral fat level of football players regarding field position.

Variable	Measurement	Striker (S) X ± SD Med (Min–Max)	*p*-Value	Midfielder (M) X ± SD Med (Min–Max)	*p*-Value	Defender (D) X ± SD Med (Min–Max)	*p*-Value	Goalkeeper (G) X ± SD Med (Min–Max)	*p*-Value	Position *p*-Value
Dominant arm	1	0.25 ± 0.1_0.25 (0.2–0.3)	0.88; NS	0.19 ± 0.1_0.2 (0.1–0.2)	0.2; NS	0.28 ± 0.17_0.35 (0.1–0.4)	0.13; NS	0.22 ± 0.16_0.2 (0.1–0.2)	0.04; 4 vs. 6 *	0.58; NS
	2	0.23 ± 0.08_0.25 (0.2–0.3)		0.19 ± 0.13_0.2 (0.1–0.2)		0.28 ± 0.15_0.3 (0.1–0.4)		0.22 ± 0.16_0.2 (0.1–0.2)		0.32; NS
	3	0.25 ± 0.1_0.25 (0.2–0.3)		0.19 ± 0.08_0.2 (0.1–0.2)		0.28 ± 0.14_0.3 (0.15–0.4)		0.2 ± 0.17_0.1 (0.1–0.2)		0.22; NS
	4	0.25 ± 0.1_0.25 (0.2–0.3)		0.2 ± 0.1_0.2 (0.1–0.2)		0.31 ± 0.16_0.4 (0.15–0.4)		0.18 ± 0.18_0.1 (0.1–0.1)		0.16; NS
	5	0.23 ± 0.15_0.2 (0.1–0.3)		0.21 ± 0.13_0.2 (0.1–0.2)		0.3 ± 0.15_0.4 (0.15–0.4)		0.22 ± 0.22_0.1 (0.1–0.2)		0.45; NS
	6	0.27 ± 0.1_0.2 (0.2–0.4)		0.23 ± 0.15_0.2 (0.1–0.3)		0.3 ± 0.15_0.4 (0.15–0.4)		0.3 ± 0.22_0.2 (0.2–0.2)		0.52; NS
Assisting arm	1	0.23 ± 0.08_0.25 (0.2–0.3)	0.71; NS	0.21 ± 0.1_0.2 (0.1–0.2)	0.12; NS	0.29 ± 0.18_0.3 (0.1–0.4)	0.39; NS	0.26 ± 0.19_0.2 (0.2–0.2)	0.07; NS	0.72; NS
	2	0.22 ± 0.08_0.2 (0.2–0.3)		0.19 ± 0.12_0.2 (0.1–0.3)		0.28 ± 0.15_0.3 (0.1–0.4)		0.24 ± 0.15_0.2 (0.2–0.2)		0.48; NS
	3	0.23 ± 0.1_0.2 (0.2–0.3)		0.19 ± 0.07_0.2 (0.1–0.2)		0.28 ± 0.14_0.3 (0.15–0.4)		0.22 ± 0.16_0.2 (0.1–0.2)		0.31; NS
	4	0.25 ± 0.08_0.2 (0.2–0.3)		0.2 ± 0.1_0.2 (0.1–0.2)		0.31 ± 0.15_0.35 (0.15–0.4)		0.22 ± 0.16_0.2 (0.1–0.2)		0.23; NS
	5	0.23 ± 0.15_0.2 (0.1–0.3)		0.23 ± 0.12_0.2 (0.1–0.3)		0.3 ± 0.15_0.4 (0.15–0.4)		0.26 ± 0.26_0.1 (0.1–0.3)		0.57; NS
	6	0.27 ± 0.08_0.25 (0.2–0.3)		0.23 ± 0.13_0.2 (0.1–0.3)		0.31 ± 0.16_0.4 (0.15–0.4)		0.34 ± 0.26_0.2 (0.2–0.3)		0.48; NS
Trunk	1	4.12 ± 1.14_4.35 (3.1–5)	0.75; NS	3.28 ± 1.14_2.9 (2.6–3.7)	0.0002; 2 vs. 6 ***	4.75 ± 1.82_4.95 (3–6)	0.004; 2 vs. 6 **	4.72 ± 1.26_4.4 (3.8–4.9)	0.57; NS	0.05; NS
	2	3.87 ± 1.11_4.1 (2.9–4.9)		3.14 ± 1.29_2.8 (2.2–3.8)		4.4 ± 1.77_4.85 (3.15–5.55)		4.76 ± 1.3_4.4 (3.8–5.4)		0.08; NS
	3	3.9 ± 1.25_3.95 (3.1–5)		3.1 ± 1.2_2.9 (2.5–3.6)		4.6 ± 1.62_4.8 (3.45–5.95)		4.8 ± 1.47_4.3 (3.6–5.7)		0.03; *p* < 0.05 M vs. D *
	4	4.02 ± 0.89_3.95 (3.3–4.9)		3.35 ± 1.15_3.1 (2.6–3.6)		4.62 ± 1.81_4.95 (3.75–6)		4.66 ± 1.37_4.1 (3.7–5)		0.1; NS
	5	4.03 ± 1.44_3.8 (3.2–4.8)		3.5 ± 1.38_3.3 (2.5–4.1)		4.7 ± 1.91_5.25 (3.7–6.1)		4.9 ± 1.88_4.2 (4.1–4.5)		0.15; NS
	6	4.03 ± 0.99_4.1 (3–5)		3.78 ± 1.41_3.2 (2.8–4.3)		5.08 ± 1.92_5.7 (3.85–6.6)		5.2 ± 2.2_4.3 (4.3–4.5)		0.14; NS
Dominant leg	1	1.37 ± 0.24_1.4 (1.2–1.6)	0.56; NS	1.17 ± 0.21_1.1 (1.1–1.3)	0.0001; 2 vs. 6 * 3 vs. 6 **	1.39 ± 0.39_1.4 (1.05–1.75)	0.003; 2 vs. 6 ** 3 vs. 6 *	1.44 ± 0.32_1.3 (1.3–1.4)	0.26; NS	0.19; NS
	2	1.28 ± 0.24_1.35 (1.1–1.5)		1.15 ± 0.27_1.1 (1–1.3)		1.33 ± 0.37_1.45 (1.05–1.65)		1.42 ± 0.31_1.4 (1.2–1.5)		0.27; NS
	3	1.32 ± 0.31_1.35 (1.2–1.5)		1.12 ± 0.23_1.1 (1–1.2)		1.35 ± 0.35_1.4 (1.1–1.65)		1.44 ± 0.36_1.3 (1.2–1.6)		0.11; NS
	4	1.35 ± 0.22_1.4 (1.2–1.5)		1.2 ± 0.24_1.2 (1–1.3)		1.36 ± 0.39_1.45 (1.15–1.65)		1.38 ± 0.31_1.3 (1.2–1.4)		0.48; NS
	5	1.33 ± 0.32_1.2 (1.2–1.5)		1.23 ± 0.3_1.2 (1–1.4)		1.38 ± 0.42_1.55 (1.15–1.7)		1.46 ± 0.44_1.3 (1.2–1.5)		0.61; NS
	6	1.33 ± 0.23_1.3 (1.1–1.6)		1.28 ± 0.31_1.2 (1.1–1.4)		1.47 ± 0.42_1.65 (1.2–1.8)		1.54 ± 0.49_1.4 (1.3–1.4)		0.33; NS
Assisting leg	1	1.35 ± 0.23_1.4 (1.2–1.5)	0.83; NS	1.17 ± 0.21_1.1 (1.1–1.3)	0.0001; 2 vs. 6 * 3 vs. 6 **	1.37 ± 0.36_1.4 (1.05–1.7)	0.004; 2 vs. 6 * 3 vs. 6 **	1.44 ± 0.32_1.3 (1.3–1.4)	0.18; NS	0.18; NS
	2	1.28 ± 0.24_1.35 (1.1–1.5)		1.13 ± 0.27_1.1 (0.9–1.3)		1.31 ± 0.38_1.4 (1.05–1.6)		1.4 ± 0.31_1.4 (1.2–1.4)		0.3; NS
	3	1.32 ± 0.31_1.35 (1.2–1.5)		1.12 ± 0.23_1.1 (1–1.2)		1.34 ± 0.33_1.4 (1.1–1.65)		1.42 ± 0.36_1.3 (1.2–1.5)		0.13; NS
	4	1.33 ± 0.22_1.35 (1.2–1.5)		1.19 ± 0.25_1.2 (1–1.3)		1.34 ± 0.37_1.45 (1.15–1.6)		1.38 ± 0.31_1.3 (1.2–1.4)		0.44; NS
	5	1.33 ± 0.32_1.2 (1.2–1.5)		1.23 ± 0.29_1.2 (1–1.4)		1.36 ± 0.4_1.55 (1.15–1.65)		1.46 ± 0.44_1.3 (1.2–1.5)		0.58; NS
	6	1.32 ± 0.23_1.25 (1.1–1.6)		1.28 ± 0.32_1.2 (1.1–1.5)		1.44 ± 0.42_1.65 (1.2–1.75)		1.54 ± 0.49_1.4 (1.3–1.4)		0.34; NS
Visceral fat level	1	2.17 ± 0.98_2.5 (1–3)	0.83; NS	1.87 ± 1.19_1 (1–2)	0.07; NS	2.92 ± 1.73_3 (1–4)	0.05; NS	2.6 ± 0.89_2 (2–3)	0.12; NS	0.27; NS
	2	2 ± 0.89_2 (1–3)		1.67 ± 0.98_1 (1–2)		2.67 ± 1.3_3 (1.5–3.5)		2.4 ± 1.14_2 (2–3)		0.15; NS
	3	1.83 ± 0.75_2 (1–2)		1.67 ± 0.98_1 (1–2)		2.75 ± 1.36_3 (1.5–4)		2.6 ± 1.34_2 (2–4)		0.11; NS
	4	1.83 ± 0.75_2 (1–2)		1.67 ± 0.98_1 (1–2)		2.83 ± 1.34_3 (2–3.5)		2.4 ± 1.14_2 (2–3)		0.07; NS
	5	2 ± 1.1_2 (1–2)		1.87 ± 1.19_1 (1–2)		2.92 ± 1.31_3 (2–4)		2.8 ± 1.92_2 (2–3)		0.15; NS
	6	2.17 ± 0.98_2.5 (1–3)		2.07 ± 1.33_2 (1–3)		3.25 ± 1.54_4 (2–4.5)		3.4 ± 1.52_3 (3–3)		0.09; NS

X = average; SD = standard deviation; Med = median; Min = minimum; Max = maximum; * = *p* < 0.05; ** = *p* < 0.01; *** = *p* < 0.001.

## Data Availability

The datasets generated during the study are available from the corresponding author on reasonable request.
